# Optimization of a Loop-Mediated Isothermal Amplification Assay as a Point-of-Care Tool for the Detection of *Wuchereria bancrofti* in Human Blood in Tana River Delta, Kenya

**DOI:** 10.1155/2021/6650870

**Published:** 2021-07-27

**Authors:** Kinyatta Nancy, Wambua Lillian, Mutahi Wilkinson, Mugasa Claire, Kamau Luna, Wachira Dorcas, Githae Rosemary, Lusweti Japheth, Ichugu Christine, Waigi Emily, Kagai Jim

**Affiliations:** ^1^Kenya Medical Research Institute, Centre for Biotechnology Research and Development, P.O. Box 54840-00200, Nairobi, Kenya; ^2^School of Biological Science, University of Nairobi, 30772-00100, Nairobi, Kenya; ^3^College of Veterinary Medicine, Animal Resources and Biosecurity, Makerere University Kampala, 7062 Kampala, Uganda

## Abstract

**Introduction:**

Accurate detection of filarial parasites in humans and vectors is essential for the implementation and evaluation of Global and National Programs to eliminate lymphatic filariasis. Immunological methods to detect infection are available; however, cross-reactivity issues have been reported in most of them. Nucleic acid-based molecular assays offer high levels of specificity and sensitivity and can be used to detect the infections.

**Methods:**

In this study, we evaluated loop-mediated isothermal amplification (LAMP) tests to amplify *Wuchereria bancrofti* DNA in patients' blood. The amplicons were tested by both pH-sensitive dyes for enhanced visual detection and agarose gel electrophoresis. A closed-tube LAMP assay was also evaluated. Cohen's Kappa statistics was used for statistical analysis of the assays. 125 patients consented for blood sampling which were used for clinical analysis of LAMP assays with the PCR method used as the “gold standard.”

**Results:**

The sensitivity of the evaluated *Wuchereria bancrofti* LAMP was 92.3%, with a specificity of 97.3% and kappa statistics value of 0.84, which is in a strong agreement.

**Conclusion:**

In this study, LAMP assays coupled with fluorescence dye detection have been found to be suitable for diagnosis and monitoring of *Wuchereria bancrofti* infections in the Kenyan population.

## 1. Introduction

### 1.1. Background Information and Literature Review

Lymphatic filariasis (LF) is a chronic parasitic disease of public health and socioeconomic significance in tropical and subtropical countries. It is caused by the filarial worms of *Wuchereria bancrofti*, *Brugia malayi*, or *B*. *timori* species. This debilitating mosquito-borne nematode infection had been earmarked for elimination by the year 2020 [1]. The disease is transmitted by five genera of mosquitoes, *Culex*, *Aedes*, *Anopheles*, *Mansonia*, and *Ochlerotatus* [2], making it the most common vector-transmitted parasitic infection after malaria [3, 4]. It is the second leading cause of permanent and long-term disability worldwide after eye blindness [5]. Acute and chronic morbidity resulting from lymphatic filariasis has affected 120 Million people living in 81 endemic countries with 1.34 billion people at risk of developing the infections [6]. In 2000, the Global Program to Eliminate LF (GPELF) was launched [7] and it was estimated that 5–6 rounds of mass drug administration (MDA) with ivermectin or diethylcarbamazine and albendazole were required to eliminate the disease [5]. In 2018, 893 million people in 49 countries were living in areas that required preventive chemotherapy to stop the spread of the infections [8].

Early and accurate diagnosis of *Wuchereria bancrofti* causing *bancroftian filariasis* is a key factor in the effort to eliminate filariasis [9]. The diagnosis has relied on the detection of microfilariae in blood specimen and in mosquitoes vectors [9]. Point-of-care diagnosis of lymphatic filariasis is largely based on microscopic examination of blood collected at night (2200 pm–2000 am) [10]. The immunochromatographic test (ICT) is a rapid format which detects specific circulating filarial antigen in *bancroftian filariasis* [11, 12]. Immunochromatographic test (ICT) is regarded by the WHO as the “gold standard” for diagnosis of lymphatic filariasis [11]. It has a number of limitations which include high cost and inconsistent availability and also detects antigen even after clearance of the worms on treatment [11]. Microfilariae antibody detection for *brugian filariasis* is available in clinical settings [12, 13]. However, the antibody tests indicate exposure rather than active infection [14] and do not distinguish between *bancroftian* and *brugian filariasis* [15–17]. Following the implementation of the control programs, monitoring is necessary to determine the endpoint of treatment, with continued surveillance being required to identify areas of ongoing transmission or recrudescence [11, 16]. These activities and overall management of MDA programs can be achieved efficiently by the use of accurate, sensitive, specific, and relatively cheap diagnostic tools suitable for point-of-care application.

Molecular-based amplification methods such as polymerase chain reaction (PCR) are proven technologies with high sensitivity and specificity [18–20] for the detection of microfilariae. These methods have not been widely used in remote settings because of the high cost and complexity of the procedures requiring trained personnel and sophisticated equipment [21]. Several PCR-based methods have been used to amplify DNA in blood from *B. malayi*- and *B. timori*- [22] and *W. bancrofti*-infected patients [18, 23, 24]. Molecular monitoring of insect vectors by PCR is also the preferred method for xenodiagnoses that has been used extensively for *W. bancrofti* [25, 26] and *B. malayi* [27, 28]. An alternative to PCR are the isothermal amplification techniques which amplify DNA at a relatively constant temperature; thus, a simple water bath or a heat block can be used [29–33].

Loop-mediated isothermal amplification (LAMP) is a unique and novel amplification method with high specificity and sensitivity able to discriminate between single-nucleotide differences [29]. It is characterized by the use of six different primers specifically designed to recognize eight distinct regions on a target gene, with amplification only occurring if all primers bind and form a product [29]. In the past, LAMP has been successfully applied for the rapid detection of both DNA and RNA viruses such as the West Nile [34] and SARS viruses [30]. Parasitologists have adapted the LAMP approach for the detection of several parasitic diseases including the human parasites *Entamoeba* [35], *Trypanosoma* [36], *Taenia* [37], *Plasmodium* [38], and *Cryptosporidium* [39]. Other parasite detection assays include the animal parasites *Theileria* and *Babesia* [40, 41]. LAMP has also been developed for the identification of vector mosquitoes carrying Plasmodium and *Dirofilaria immitis* parasites [42]. Most of these studies have brought to light the many advantages of this method over the common PCR technique. In addition, LAMP results can readily be assessed by white precipitate formation from large amounts of pyrophosphate ions as a by-product generated during the reactions, and also, the results can be assessed by the naked eyes or by adding a fluorescence dye [36, 43]. The applicability of LAMP to field surveys and hence as a diagnostic and mapping tool for filarial infections was first demonstrated by detecting *Dirofilaria* in wild-caught mosquitoes [44]. Later, Takagi et al. [32] found out that the LAMP method was able to detect *W. bancrofti* DNA in human blood and mosquito pools and a potential tool for field applications with more validation studies.

Due to limitations of most of the diagnostic methods highlighted, for a precise reveal of the distribution of filarial infections, novel diagnostic methods that are simple, rapid, sensitive, and reliable are required. In consideration of these points, this study was to evaluate and validate LAMP as a molecular isothermal amplification assay for diagnosis of *Wuchereria bancrofti* to aid in monitoring the prevalence of the disease in Kenyan endemic areas.

## 2. Materials and Methods

### 2.1. Study Site

**Figure 1:**
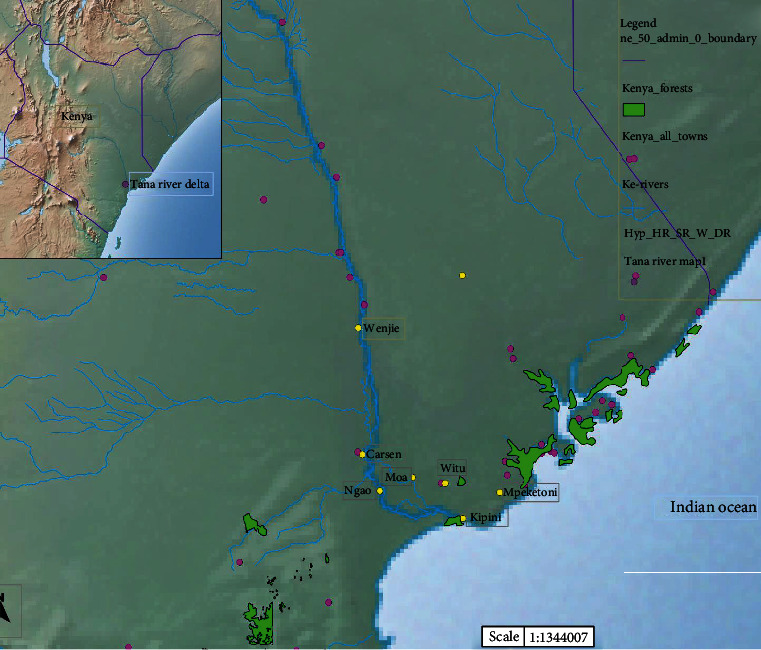
Map of Tana River Delta in Tana River County, Kenya. Source: map generated by Jacob Mueti.

Samples for this study were collected from Tana River Delta in Tana River County, Kenya (Figure 1, showing a map of the Tana River Delta). This region is one of the endemic areas considered to have high prevalence (22.2%) [45] where MDA Programs started in 2011 [46]. Tana River Delta was curved off from Tana River district in 2007. It has 3 divisions Kipini, Garsen, and Tarasaa with an area of 16013 km^2^. Tana River County has a population of 250000 with 134000 in Tana River district according to the 2009 census [47]. The rainfall ranges between 220 and 900 mm per year, while the average temperature is 30°C. The altitude ranges between 0 and 200 m. The major ethnic groups are the Pokomo, who are subsistent famers and practice fishing, and Orma and Wardey who are predominantly nomadic.

### 2.2. Ethical Clearance

The permission to carry out this study was sought from the Kenya Medical Research Institute Scientific and Ethical Review Unit (SERU), protocol number SSC. 2802. Participants had been requested to consent (adults) or assent (children) to participate in the study by giving the blood sample to be used in this study.

### 2.3. LAMP Primer Designing

The primers used to perform the present assay were those previously identified by Takagi et al. [32]. The target was 18s rRNA species-specific regions (Ssp1), a highly repeat gene on the *W. bancrofti* complete sequence of accession number (AY297458), yielding 188 base pairs. The diagrammatic representation of sets of primers targeting *Wuchereria bancrofti* mitochondria DNA is as shown in Table 1.

### 2.4. LAMP Primer Specificity Testing

Before optimizing the LAMP assays, the specificity of the outer primers (F3 & B3) was tested by conventional PCR. The expectations here were that primers specific for *W. bancrofti* detection would show amplification only on positive controls and with no amplification on negative controls. The positive controls used here were *W. bancrofti* commercial controls and a known positive specimen as earlier determined by conventional PCR with NV1 and NV2 Ssp1 repeat sequence primers. The positive controls had been sequenced earlier with accession numbers MK471341 and MK 471347 on the gene bank. The negative controls included blood specimen from the nonendemic area, blank Master Mix, and the PCR water as initially confirmed by PCR.

### 2.5. Sample Processing and Amplification

Blood samples were collected from consenting patients into well-labeled vacutainers. Samples were subjected to DNA extraction by alcohol precipitation method as described by Datta et al. [48] with minor modifications. The quantity and quality of the extracted DNA was determined by measuring A260 and the ratio of A260/A280 on a NanoDrop ND-1000 spectrophotometer (Thermo Scientific, USA). The DNA was amplified by LAMP and polymerase chain reaction assays for comparison. Detection of the amplicons was by agarose gel electrophoresis and 1 : 10 SYBR Green 1 dye or a florescence dye.

#### 2.5.1. Polymerase Chain Reaction (PCR)

The PCR was performed as originally reported by Nicolas and Plichart [18]. Two primers as identified by Zhong and colleagues [49] as NV-1 and NV-2 were used. The PCR reaction mix contained 12 *μ*l of 10x Bioline buffer with Mgcl2 and dNTPs, 5 pmol/*μ*l of NV1 and NV2 primers each, 5 *μ*l genomic DNA template, and water to top up the reaction volume to 25 *μ*l. The PCR reaction was run in a 96-well GeneAmp® PCR system 9700 with conditions consisting of a single step of 95°C for 5 minutes, 40 cycling step of 94°C for 30 seconds, 54°C for 45 seconds, 72°C for 30 seconds, and a final extension step of 72°C for 10 minutes. The PCR products were size fractioned on 2.0% agarose gel stained with Ethidium bromide. Agarose gel electrophoresis was run at 80 V for 60 minutes and bands visualized under UV light using a gel documentation system (EZ Imager, Bio-Rad, CA). Positive control and negative controls were included in every run to ensure specificity and validity of the results. The expected *W. bancrofti* size band was 188 base pairs and this was measured against a 100 base pair molecular weight marker.

#### 2.5.2. LAMP Optimization

Optimization of the LAMP assay was done by varying the reaction temperatures and time, as well as varying the concentrations of LAMP primer sets of forward and backward outer primers (F3 and B3) and forward and backward inter primers (FIP and BIP). The final assay-optimized conditions were as follows: the final reaction mixture of 25 *μ*l contained primers (40 pmol of FIP and BIP and 5 pmol of F3 and B3 outer primers), DNA polymerase, 8 units of Bst I large fragment (Meridian Bioscience®), 1 mM dNTPs, 0.8 M betaine, and 1x reaction buffer (containing 20mMTris-HCl, pH 8.8, 10 mM KCl, 10 mM (NH4) 2SO4, 8mMMgS4, and 1% Tween 20). The reaction was incubated at varying temperature ranging from 60°C to 65°C on a heat block for time between 30 minutes and 60 minutes. During the optimization process, DMSO 7.5% as per assays by Wang et al. [50] was added to reduce false-positive results due to primer dimers.

To make LAMP amplification more applicable in a field setup, the Commercial Isothermal Amplification kit was used from Eiken Chemical Co. (Tochigi, Japan). Direct detection of amplicons in a reaction tube was done by direct observation of the reaction with the unaided eye for the color change after addition of 1 *μ*l of 1 : 10 SYBR Green I dye (Invitrogen, Carlsbad, CA) to the amplicon. The DNA product was also visualized under ultraviolet light at 320 nm after electrophoresis on 2% standard agarose gel for 60 minutes at 80 V and then photographed for records.

#### 2.5.3. Closed-Tube Detection: Lyophilized LAMP Reaction Buffers

To further assess LAMP application in a field setup, closed-tube detection to reduce aerosol pollutants and cross-pollutant with SYBR Green 1 for detection was used. The reaction kit—Loopamp DNA amplification reagent—contains all essential reaction components (reaction buffers and dNTPs) in a dried form on the cap of each reaction tube. For the reaction, only the specific primers and the DNA template were added and incubated at appropriate conditions. The Commercial Isothermal Amplification kit was used from Eiken Chemical Co. (Tochigi, Japan). Direct detection of amplicons in a reaction tube was done by direct observation of the reaction with the unaided eye for turbidity in the presence of amplified *Wuchereria bancrofti* DNA and viewed under UV light for any color change in addition to 1 *μ*l of 1 : 10 SYBR Green I dye (Invitrogen, Carlsbad, CA).

### 2.6. Analytical LAMP Specificity Testing

The specificity of the LAMP assay was carried out using *W. bancrofti*-specific primers to amplify DNA of other related parasites including *Brugia malayi* [27], *Schistosoma mansoni*, *Plasmodium falciparum*, *Trichuris trichiura*, *Echinococcus granulosus*, and mosquito vectors *Anopheles gambiae* [12, 14, 23, 27–29, 32, 38]. These parasites had been tested by PCR using specific primers for each parasite target region; their sequences are found in the gene bank.

### 2.7. Analytical LAMP Sensitivity Testing

To determine the sensitivity of the LAMP assay by establishing the lower detection limit, the genomic DNA concentration was determined using a NanoDrop 1000 and a 10-fold serial dilution of the DNA done. The successive serially diluted DNA was then amplified with LAMP primers to determine assay sensitivity. Also, to compare the sensitivity with the PCR sensitivity, the same dilutes were run by PCR.

### 2.8. Clinical LAMP Sensitivity and Specificity Testing

Clinical testing of the assay sensitivity and specificity was done; 125 clinical specimens from participants were tested on both PCR and LAMP assay. The LAMP assay sensitivity, specificity, and Kappa statistics values were determined by the 2 × 2 contingency table.

## 3. Study Results

The results described here show the amplification of *W. bancrofti* DNA using LAMP primers targeting the Ssp 1 repeat sequence. LAMP primers used in this study consistently amplified the Ssp 1 repeat sequence in a ladder like having multiple bands of different sizes under isothermal amplification conditions of 63°C for 60 minutes. The test results indicated that the developed LAMP assay could successfully amplify *W. bancrofti* in a patient's blood specimen. The amplified products were detected either by use of 1 : 10 SYBR Green 1 dye by observing the color change or by agarose gel electrophoresis. A closed tube with lyophilized reaction buffers was also used to access their use in minimizing contamination and the ease of field applicability.

### 3.1. LAMP Amplicon Detection by Florescent Dye

**Figure 2:**
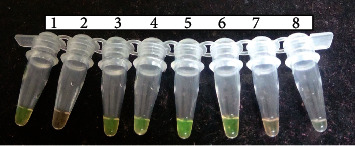
LAMP florescence dye detection (open tubes). Tube no. 1: *W. bancrofti* DNA-positive control; 2: negative control; 3, 4, 5, and 6: positive specimens; 7 and 8: negative specimens. Green color indicates that W*uchereria bancrofti* DNA was present and was amplified (positive). Orange color means that there was no *W. bancrofti* DNA and hence no amplification that took place (negative).

Before the incubation, the color of the reaction mix was orange. In the presence of a positive LAMP DNA amplicon, the color in the reaction tube changed from orange to green and remained orange in the absence of amplicons (Figure 2).

### 3.2. Gel Representation of the LAMP Amplicon Results

**Figure 3:**
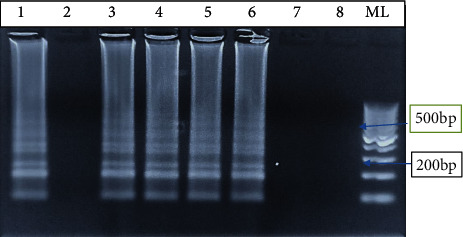
LAMP amplification results on agarose gel. Lane 1: positive control of *W. bancrofti* DNA; lane 2: negative control; lanes 3, 4, 5, and 6: positive specimens; and lanes 7 and 8: negative results. Lane ML is the molecular weight marker of 100 bp.

LAMP-positive results are indicated by a stream of ladder-like light bands at different lengths, indicating the presence of *W. bancrofti* DNA in the specimen. There are no observable bands in the absence of an amplicon as shown in Figure 3.

### 3.3. LAMP Reaction in Closed Tubes: “Ready-to-Use Buffers”

**Figure 4:**
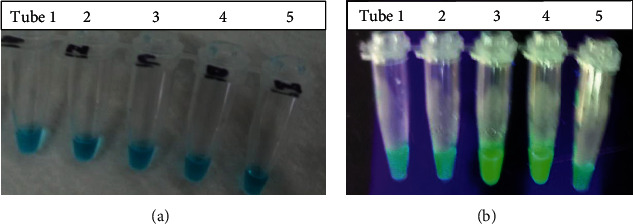
(a) LAMP reaction mix before amplification. (b) LAMP mix reaction after amplification. (a) All reaction mix in tubes 1–5 was blue in color before incubation. On incubating the reaction mix at 63°C for 60 minutes, the results were as shown in (b) on the addition of 1 : 10 SYBR Green dye and viewed under a UV Light or under sunlight. Tubes 1 and 2 remained blue in color indicating the absence of *Wuchereria bancrofti* DNA (negative specimen), tube 3 turned green in color indicating positive *W. bancrofti* DNA amplification (positive specimen), tube 4 contained *W. bancrofti* DNA-positive control which turned green in color, and tube 5 was a negative control which remained blue in color.

The use of closed tubes containing ready-to-use reaction buffers showed color change from original blue before amplification as shown in Figure 4(a) to light-green in the presence of *W. bancrofti* DNA in Figure 4(b) on amplification. Tubes 3 & 4 on amplification at 63°C for one hour turned green while tubes 1, 2, & 5 remained blue indicating the absence of *W. bancrofti* DNA as viewed under a UV light. The green color indicates a positive specimen while a negative specimen remains blue even after amplification.

#### 3.3.1. Analytical LAMP Sensitivity and Specificity Evaluation

**Figure 5:**
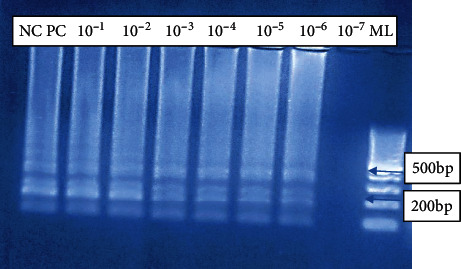
LAMP sensitivity testing on gel electrophoresis. Lane NC represents negative control; lane PC represents positive control; lanes 10^−1^–10^−6^ represented serial dilutions of DNA extracts for testing the assay sensitivity. 10^−1^–10^−6^ showed amplification of *W. bancrofti* as bands appeared at different lengths indicating that our LAMP assay was sensitive and could detect to up to 1/1000000 (1/10^6^) DNA copies of the parasites in the diluent, while no band was observed in 1/10000000 (1/10^7^) copies in the diluent.

**Figure 6:**
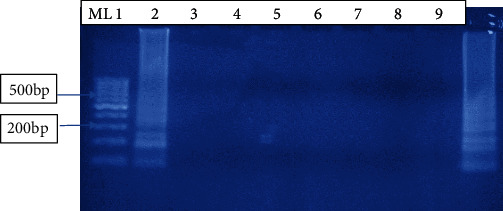
LAMP specificity testing on agarose gel electrophoresis. Lane ML is a molecular weight marker 100 bp; lane 1: *W. bancrofti* DNA-positive specimen; lanes 2–7: non-*W. bancrofti* DNA of other parasites which did not show any amplification; lane 2: *Brugia Malayi*; lane 3: *Anopheles gambiae*; lane 4: S*chistosoma mansion*; lane 5: *Plasmodium falciparum*; lane 6: *Trichuris trachura*; lane 7: *Echinococcosis granulosus*; lane 8: negative control; lane 9: *W. bancrofti* DNA-positive control.

Our LAMP assay showed a sensitivity limit of 10^−6^ which was equivalent to (1/1000000) DNA copies of the parasites in the diluent, while no band was observed in 1/10000000 (1/10^7^) copies in the diluent as shown in Figure 5. The assay showed a great specificity because it could only amplify *W. bancrofti* DNA when tested alongside other parasites. *W. bancrofti* DNA showed a stream of bands at different sizes, and the absence of *W. bancrofti* DNA showed no bands (Figure 6).

#### 3.3.2. PCR Assay Sensitivity and Specificity Testing

**Figure 7:**
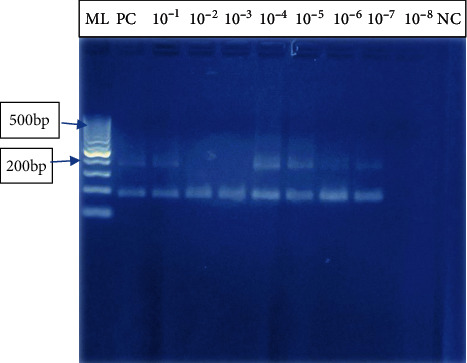
PCR sensitivity testing on gel electrophoresis. Lane ML: molecular marker 100 bp; lane PC: positive control, *W. bancrofti* DNA; lanes 10^−1^–10^−7^: serial dilutions of DNA extracts for testing the assay sensitivity; lane 10^−8^: no amplification; lane NC: represents negative control; 10^−1^–10^−7^ showed amplification of *W. bancrofti* as bands appeared just below the 200 bp as the expected band size was 188 bp. This showed that PCR assay was sensitive and could detect to up to 1/1000000 (1/10^7^) copies of parasite DNA in the diluent, while no band was observed in 1/10000000 (1/10^8^) copies in the diluent.

When the same diluent used for LAMP sensitivity testing was used to run PCR, it was found that PCR could detect up to 10^−7^ indicating that it was a little bit more sensitive as compared to LAMP (Figure 7). There were no bands on DNA from non-*W. bancrofti* species.

### 3.4. Time-Dependent Specificity Testing Using Florescence Dye Detection

The duration of amplification time was evaluated to find out whether it has any impact on amplification; the results obtained within 30 to 60 minutes of incubation were relatively the same. There was no much color change even with the increased time for amplification at the same temperature as shown in Table 2.

### 3.5. Statistical Analysis of LAMP Results with PCR (Gold Standard) Results

Clinical sensitivity and specificity of LAMP was done by examining 125 patient's blood. Out of 125 samples examined, 13 were positive by PCR and 15 samples were positive by LAMP. A total of 112 samples were confirmed to be negative with PCR whereas LAMP confirmed a total of 110 specimens as negative. With respect to sensitivity, LAMP had a sensitivity of 92.3% and a specificity of 97.3% at 95% confidence interval and a power of 1. When Cohen's Kappa coefficient was determined, the value was 0.84, suggesting that there was an exceptional agreement between the two techniques.

## 4. Discussion

Accurate, specific, sensitive, affordable, and less-sophisticated diagnostic tools are needed for point-of-care detection and prompt initiation of treatment and prevention of *Wuchereria bancrofti*. The intensified control programs have significantly led to low human infection and low transmission rates in vectors. This has led to the need for accurate and specific tools for identifying endemic areas where treatment is required and when the transmission limit level is attained in order to declare an endemic region free of filariasis.

LAMP is a novel DNA amplification method that allows reactions to occur under isothermal conditions unlike PCR which involves cycles of varying temperatures by a specialized equipment [36]. LAMP has been developed and optimized for viruses and a number of parasites [30, 34–42]. However, studies for its application in the field are scanty. Advantages of LAMP reactions are that the use of the four to six primers—FIP, BIP, F3, B3, and/or 2 loop primers—to recognize six to eight different regions of interest in a sequence makes LAMP to have a higher specificity to the reaction compared to conventional PCR methods [36]. Another advantage using LAMP is based on the fact that the amplification from stem-loop structures leads to accumulation of large amounts of products of varying lengths. Ultimately, this makes detection of amplified DNA much easier by visual observations eliminating the need for postamplification detection by gel electrophoresis.

In this study, we evaluated the performance of LAMP assays in detection of *Wuchereria bancrofti*. Optimization was done by changing the primer concentrations, varying in time and temperature. The use of lyophilized reagents in a closed tube was also assessed. Detection was done by visualization through color change by use of florescence dye, 1 : 10 SYBR Green 1 and by gel electrophoresis. The color change, from orange to green, in the presence of amplified DNA showed an easy way of detecting amplicons (Figure 2) without the need for postamplification analysis by gel electrophoresis that uses ethidium bromide which is carcinogenic in nature, and minimizing its use is an important aspect of public health safety. The gel representation of the LAMP amplification results is as in Figure 3. These results were in accordance with the reports by Notomi et al. [29]. The closed tube in Figure 4(a) shows the reaction mix which is blue in color before amplification. After amplification at 63°C for 60 minutes, the results were as in Figure 4(b). In the presence of *W. bancrofti* DNA, the color changed from blue to greenish as viewed under ultraviolet light (260 nm) and remained blue in the absence of *W. bancrofti* DNA. The closed tubes had an added advantage since there was minimal handling of reaction reagents and this reduced the chances of cross-contamination. This study yielded results which were comparable to studies on the LAMP colorimetric test by Poon et al. [38], Goto et al. [51], and Poole et al. [52]. Tenfold serial dilution was done to test the LAMP sensitivity as showed in Figures 5 and 7 for LAMP and PCR, respectively. The initial concentration was 50.0 ng/*μ*l which is equivalent to 200 pg; approximately 1 (one) DNA copy was used to make the serial diluent. The clinical sensitivity of the developed LAMP was 92.3% at 95% confidence interval and with a power of 1, with 3 more specimens testing positive as compared to PCR results. Specificity of the evaluated *W. bancrofti* LAMP assays was 97.3%. Only specimen containing *Wuchereria bancrofti* species showed amplification either by color change or by gel electrophoresis as in Figure 6. Cohen's Kappa statistics showed a great agreement of 0.84 indicating that the two methods are comparable and that LAMP assays can substitute PCR in resource-limiting regions.

The *W. bancrofti* LAMP test described here shows a greater potential for use in the poorly equipped laboratories and in field setup characteristic of regions of neglected tropical disease. The detection limit for our LAMP assay was 1/10^6^ which is equivalent to 1 microfilariae per 200 *μ*l of blood (Figure 5); this gives it an advantage for use in low-prevalence and low-transmission zones.

The major challenges faced during the optimization of our *W. bancrofti* LAMP assays were the need to deal with the high rate of nonspecific amplifications that can lead to a lot of false positives. When using the opened-tube LAMP technique, there was possible cross-contamination when the lids of the reaction tubes were opened at the end of the reaction for gel electrophoresis and when adding dye for result visualization. These drawbacks faced here were similar to those reported in [36, 53–56]. To reduce false positives and false negative, DMSO at 7.5% was used in a reaction mix and this greatly improved the LAMP specificity. It was also noted that use of the closed-tube LAMP assays minimized contamination problems because of the preprepared reaction buffers in the tubes. In addition, running gel electrophoresis at the end of the LAMP reaction requires this step to be conducted in the lab which is time-consuming and therefore not suitable for on-site rapid detection.

## 5. Conclusion

We have optimized LAMP assays capable of detecting *Wuchereria bancrofti* in human blood. The assays are highly sensitive and species specific and can be used with a wide variety of DNA templates (genomic DNA, extracted DNA, boiled fresh whole blood, or blood spot samples). When closed tubes (lyophilized reagents) were applied to clinical samples, the LAMP assays were very promising and represented a powerful alternative to PCR. The LAMP method will be of benefit to global health programs aimed at eliminating filarial infections.

## 6. Recommendations

We recommend the use of closed-tube reaction with premix to avoid cross-contamination which was a major challenge throughout our study during the open-tube analysis. Proper primer designing and assay optimization are required to avoid false positives as a result of primer dimers.

## Figures and Tables

**Table 1:** LAMP and PCR primer sets used for the detection of *W. bancrofti* in this study.

Target repeat sequence	Amplification assay	Primer position	Sequence (5′……………….3′)	Purpose
Ssp 1	LAMP primers	FIP; F1_C_ + F2	CGACTGTCTAATCCATTCAGAGTG- TATCTGCCCATAGAAATAACTACG	Forward inner primer
		BIP; B1_C_ + B2	TCTGTGCTGAATTTTTGTGGATTG-CCAAACTAATTGTAAGCAGTCTT	Reverse inner primer
		F3	TTTGATCATCTGGGAACGT	Forward outer primer
		B3	AAGCACCTTAAATCTGTCAAT	Reverse outer primer
	PCR primers	NV-1	5′-CGTGATGGCATCAAAGTAGCG-3′;	Forward primer
		NV-2	5′-CCCTCACTTACCATAAGACAAC-3′.	Reverse primer

**Table 2:** Time-dependent testing.

Sample	Incubation time (minutes)		
	30-minute amplification	40-minute amplification	60-minute amplification
*W. bancrofti*-positive control	Green color	Green color	Green color
*W. bancrofti*-positive specimen (K19)	Light green color	Green color	Green color
*S. mansoni*	Orange color	Orange color	Orange color
Negative control	Orange color	Orange color	Orange color
Master Mix -Blank	Orange color	Orange color	Orange color

The light-green/green color indicated the presence of *W. bancrofti* DNA while the orange color indicates that no amplification took place.

## Data Availability

All data is included in the text.
